# Nickel-iron layered double hydroxides for an improved Ni/Fe hybrid battery-electrolyser†

**DOI:** 10.1039/d1ma00024a

**Published:** 2021-05-25

**Authors:** A. Iranzo, F. M. Mulder

**Affiliations:** Materials for Energy Conversion and Storage (MECS), Department of Chemical Engineering, Delft University of Technology Van der Maasweg 9 2629HZ Delft The Netherlands f.m.mulder@tudelft.nl

## Abstract

The transition to renewable electricity sources and green feedstock implies the development of electricity storage and conversion systems to both stabilise the electricity grid and provide electrolytic hydrogen. We have recently introduced the concept of a hybrid Ni/Fe battery-electrolyser (battolyser) for this application^1^. The hydrogen produced during the Ni/Fe cell charge and continued electrolysis can serve as chemical feedstock and a fuel for long-term storage, while the hybrid battery electrodes provide short term storage. Here, we present Ni–Fe layered double hydroxides (NiFe-LDHs) for enhancing the positive electrode performance. The modified Ni(OH)_2_ material capacity, high rate performance and stability have been tested over a large range of charge rates (from 0.1C to 20C) over 1000 cycles. The Ni–Fe layered double hydroxides allow the capacity per nickel atom to be multiplied by 1.8 in comparison to the conventional β-Ni(OH)_2_ material which suggests that the nickel content can be reduced by 45% for the same capacity. This reduction of the nickel content is extremely important as this presents the most costly resource. In addition, Fe doped Ni(OH)_2_ shows improved ionic and electronic conductivity, OER catalytic activity outperforming the benchmark (Ir/C) catalyst, and long term cycling stability. The implementation of this Fe doped Ni(OH)_2_ material in the Ni/Fe hybrid battery-electrolyser will bring both electrolysis and battery function forward at reduced material cost and energy loss.

## Abstract 2

The substitution of iron by nickel in Ni_0.8_Fe_0.2_-layered double hydroxides improves battery storage charteristics as well as oxygen evolution efficiency for electrolysis.

## Graphical abstract

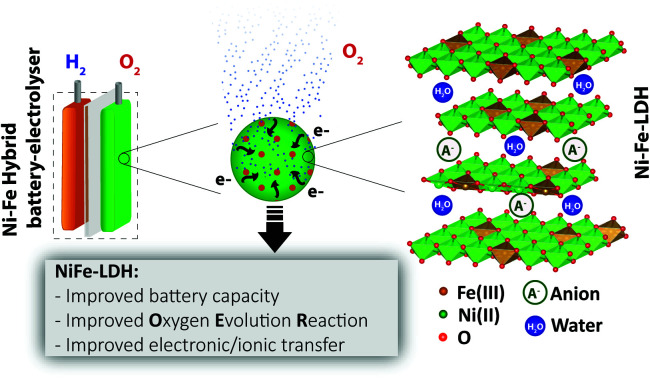

## Introduction

Annual electricity generation from renewable energy sources is growing rapidly. Renewable electricity sources represent 26% of the world's electricity today and according to the International Energy Agency (IEA) it is expected to reach 30% by 2024.^2^ IEA expects solar energy to play the largest role in the rise of the renewable energy share. Due to their inherent intermittency, renewable energies can have a serious impact on the electricity market in times of over and under supply. This can lead to curtailment or decreased or even negative electricity prices caused by a serious mismatch of supply and demand. Germany's electricity prices have dropped below zero 22 times between 2011 and 2018. More recently in April 2020, during the Coronavirus crises, hourly day-ahead power in Europe dropped to negative prices for 6 consecutive weeks. Besides, electricity prices increase when a lack of renewable generation occurs and more and more fossil power backup is phased out. These examples prove the necessity for developing large scale energy storage systems to stabilise the electricity grid by load balancing diurnal and seasonal cycles, especially when the supply of renewables starts to outgrow the instantaneous demand.

**Fig. 1:**
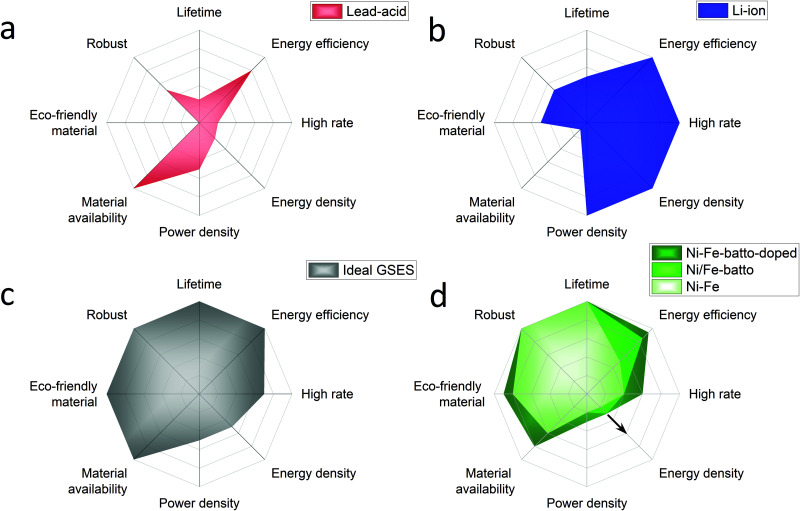
Performance radar charts of batteries conventionally used for grid-stabilisation application: (a) lead acid battery, (b) lithium ion battery, compared to (c) an ideal GSESS and to (d) the Ni/Fe battery used as conventional battery (light green), as battolyser (middle green), as modified battolyser with Ni–Fe layered double hydroxide as the positive electrode material (dark green). For each criterion the external line of the charts represents the best performance reached by rechargeable batteries. From Ni/Fe battery to Ni/Fe battolyser the energy efficiency is increased because of the hydrogen utilisation and the energy density is increased because of higher utilisation of the electrode material.^11^ The integration of α-Ni_1−*x*_Fe_*x*_(OH)_2_ material, in dark green, will induce an increased performance on materials cost, high rates and energy efficiency. If the electrolysis is included the energy handled, at the same infrastructure footprint, increases. Depending on the operational hours as battery or electrolyser and the way H_2_ is stored (gaseous, liquid, or as *e.g.* liquid ammonia) higher energy densities can be realised indicated by the arrow.

Hydrogen produced by electrolysis is a promising solution for long-term electricity storage. However, it suffers from a lower roundtrip efficiency than other storage technologies.^3^ Rechargeable batteries, with their high round trip efficiency, scalability and flexibility are particularly good candidates to balance the electricity grid in a short timescale.^4^ In the previous century several battery systems have been developed but only a few have been demonstrated in large-scale applications, mainly lead-acid and lithium ion batteries. Traditionally lead-acid had the biggest share but in 2017 Li-ion accounted for 90% of the large-scale battery storage.^5^ To understand why, an overview of these batteries performances is presented in Fig. 1a and b with radar charts showing the electrochemical properties on the right half of the chart and the criteria related to the use of the battery on the left part. The lead-acid battery is a well-known battery that requires low maintenance but its main strength is its low cost. However, its deployment is limited by the limited cycle lifetime (500–800 cycles), energy density (30–50 W h kg^−1^) and toxicity of the raw materials.^6,7^ In addition the lead-acid battery suffers also from poor high rate performance with a charging time of 8–15 hours.^7^ The lithium-ion battery outclasses the lead-acid battery with a longer lifetime (>1000 high depth of discharge cycles), good high-rate performances (charging time <1 hour) and an energy and power density among the highest reported for rechargeable batteries (170–250 W h g^−1^).^6,7^ High energy and power densities are primordial for applications that require compact and light storage devices (laptops, power tools, smartphones, and electric vehicles). However, different requirements are expected for stationary energy storage applications. Energy storage systems used for this application must have extraordinarily long cycle life, be capable of high power charge and discharge for minutes, have very high energy efficiency and, above all, have low capital and lifetime costs.^8–10^ For instance the EU SET plan defined as target properties for the grid-scale battery system a lifetime of 10 000 cycles (at 80% DOD), a high energy efficiency >90% and low capital costs <150 € kW^−1^ h^−1^.^11^ Additional requirements such as enhanced safety and the reduction of the use of critical materials are also specified. Based on this description, Fig. 1c proposes a radar chart representation of the expected performance of an ideal grid-stabilisation energy storage system (GSESS). This simple representation highlights that the main advantages of the lithium-ion battery are actually not key requirements for stationary application. This is because this application can afford bulkier devices and hence lower energy and power density batteries. Other limitations to the widespread deployment of the Li-ion battery for grid-stabilisation are the material cost and its low robustness. Li-ion shows indeed a low tolerance to overcharge and deep discharge which causes thermal runaway; additional and expensive safety systems are then required for cooling the battery and to limit the battery discharge to 80%.

The rechargeable Ni/Fe alkaline battery constitutes an interesting alternative for meeting the demands of grid scale electrical energy storage systems. Although the Ni/Fe battery shows a lower energy density than the lithium-ion battery, its specific energy (50 W h g^−1^ ^12^) is still 1 to 1.5 higher than for the lead-acid battery. In addition, the Edison battery is well known for its extraordinary robustness (2000–5000 Cycles),^13^ and its tolerance to overcharge and deep discharges (performance radar chart is shown in Fig. 1d). The low cost and abundance of the raw materials required to produce Ni/Fe cells are also two important advantages of this technology. The Ni/Fe battery presents also some drawbacks such as the use of relatively expensive Ni(OH)_2_ used for the positive electrode but more importantly, the relatively low full cell energy efficiency (65–70%). This last point explains the low interest received by the Ni/Fe technology recently. The reason in part being that, when charging, the Ni/Fe forms NiOOH and metallic Fe which are known to be good catalysts for OER (oxygen evolution reaction) and HER (hydrogen evolution reaction), respectively, inducing a competitive water splitting reaction during battery charge. To overcome this efficiency issue, Mulder *et al.*^1^ proposed to use the Ni/Fe battery in an hybrid alkaline battery-electrolyser device named battolyser in which the hydrogen production is no longer seen as a side product but instead as a main electrolysis fuel product for long term storage next to battery function. Thus, the total energy efficiency is increased up to 80–90% for both applications combined, allowing Ni/Fe to compete with the Li-ion battery (90–94%) and typical electrolysers (60–80%).^14,15^ Most remarkably, as revealed by the radar representation of Ni/Fe performance in Fig. 1d, when used as a battolyser the Ni/Fe performances are getting close to the ideal grid-stabilisation energy storage. A schematic diagram of the operation principle of the Ni–Fe hybrid battery-electrolyser is provided in the ESI,† (Fig. S-1).

To improve this hybrid battery electrolyser further, efforts must be made to reduce the material cost, improve high rate performances and increase the energy efficiency further. The main cost of the active electrode material lies in the nickel hydroxide compound, while the iron is relatively low in cost. In terms of energy efficiency, the nickel electrode also stands out for its lower intrinsic conductivity and for the OER overpotentials during electrolysis; both are determining parts of the energy efficiency loss. In this context, we focus here on the development of modified active material for the nickel electrode dedicated to the double battery-electrolyser functionality.

Commercial Ni electrodes are made of β-Ni(OH)_2_ which is one of the two polymorphic forms of nickel hydroxide. The second form is α-Ni(OH)_2_ which has been considered highly promising as the next generation cathode material for Ni-based batteries due to its higher theoretical specific capacity (490 mA h g^−1^) in comparison to that of β-Ni(OH)_2_ (289 mA h g^−1^). This alpha phase consists of positively charged β-Ni(OH)_2_ layers intercalated by water molecules and anions (mostly counter-anions of the nickel salt used for the synthesis). The interlayer space is hence larger for the alpha phase (>7.6 Å) than for the beta phase (>4.6 Å) allowing a better pathway for ionic transfer. However, in the highly dehydrating alkaline electrolyte of the Ni/Fe battery (6 M KOH), the alpha phase rapidly converts to β-Ni(OH)_2_. The unique approach to stabilise α-Ni(OH)_2_ is by partial substitution of Ni^2+^ in the hydroxide layer by trivalent metal cations. Thus, the strength of the anion binding to the layer is enhanced by the increase of positive charges in the layer, allowing the stabilisation of the alpha phase. The alpha phase shows another advantage compared to β-Ni(OH)_2_. When overcharged, β-Ni(OH)_2_ easily turns to γ-NiOOH which has a higher interlayer spacing of 7 Å, resulting in a large volume expansion of the electrode, while, during charge, α-Ni(OH)_2_ also forms γ-NiOOH but starting from a similar interlayer spacing. The stabilisation of the alpha phase therefore limits the electrode dimensional changes during (dis)charge and overcharge (*i.e.* electrolysis).

In the nickel hydroxide battery literature, different cations have been considered such as Co^16–19^ to enhance conductivity, Al,^20–26^ and Zn.^27,28^ These cations were also chosen for their poisoning effect on the OER reaction.^29^ However, in this work, OER has become an asset next to the battery functionality which makes other cation choices interesting. Iron offers the advantage of a good stability at the trivalent state;^30^ as well as a high OER catalytic behaviour when combined with Ni as Fe–NiOOH.^31–35^ Indeed, recently NiFe-LDH has gained increasing attention in the water oxidation field and is now recognized as one of the most promising OER catalysts for alkaline media.^36,37^ Several studies reported that NiFe-LDH shows higher electrocatalytic activity and stability for OER in alkaline media than commercial precious metal based catalysts.^38–40^ For instance, Yan *et al.*^37^ reported the synthesis of a 3D self-supported Ni_1−*x*_-Fe_*x*_OOH/carbon fibre cloth with excellent OER activity and stability. This catalyst provides a current density of 100 mV cm^−2^ for a low overpotential of 200 mV and shows a good stability over 100 hours. However, the synthesis methods employed for these materials appear difficult to scale-up for mass production.^38^

Here we propose the synthesis of nickel-iron layered double hydroxides (NiFe-LDHs), α-Ni_1−*x*_Fe_*x*_(OH)_2_ by a simple and easily scalable one step co-precipitation method, without any additives or surfactants. The proposed α-Ni_1−*x*_Fe_*x*_(OH)_2_ material is then expected to reduce the cost of materials by increasing the storage capacity per Ni atom, to improve the high rate performance and the energy efficiency of the hybrid Ni/Fe battery by an enhanced OER activity and conductivity (without using Co), and finally to limit structural fatigue induced by lattice expansions.

## Results and discussion

### Material characterisation

**Fig. 2:**
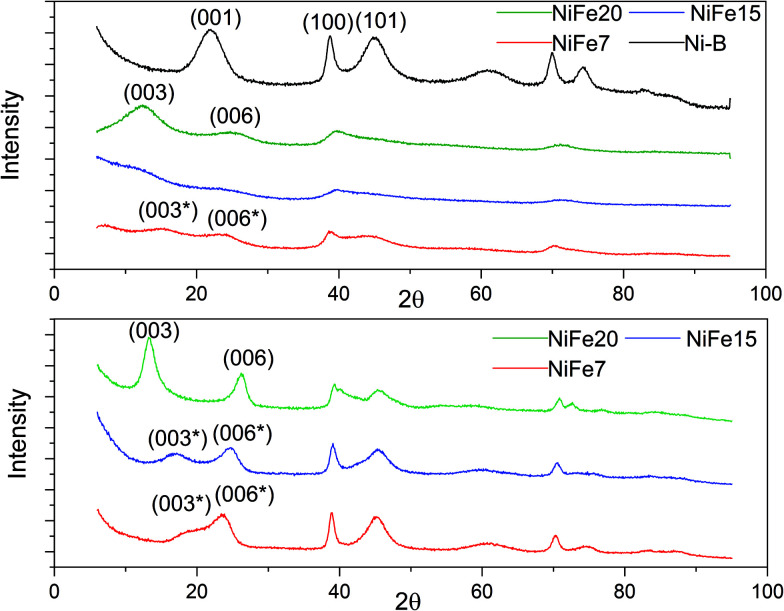
XRD of Fe doped Ni(OH)_2_ materials. Top: As prepared materials. Bottom: XRD patterns of the iron doped α-Ni(OH)_2_ after 1 month of ageing in KOH (6 M).

The XRD patterns of the as-prepared NiFe-LDH materials and a pure Ni(OH)_2_ material (synthesised following the same protocol) are shown in Fig. 2. As expected, the nickel hydroxide material prepared without iron substitution, Ni-B, presents a pure beta phase with an interlayer distance of 4.7 Å related to the *d*_001_ reflection. The NiFe-LDH samples show low crystallinity with broad and asymmetric reflections which are characteristic of a turbostratic structure often observed in the alpha phase.^41^ NiFe15 (α-Ni_1−*x*_Fe_*x*_(OH)_2_ with *x* = 0.15) and NiFe20 (α-Ni_1−*x*_Fe_*x*_(OH)_2_ with *x* = 0.20) diffractograms indeed reveal α-Ni(OH)_2_ with a rhombohedral structure (space group *R*3*m*). The diffractograms can be indexed on a hexagonal cell (Table 1) where the c-lattice parameter, reflection (003), suggests an interlayer distance (*d*_003_) of 8.64 Å for NiFe15 and 8.25 Å for NiFe20. A reduction of the interlayer distance with the concentration of iron was also observed by L. Demourgues-Guerlou *et al.*^42^ and described as a result of the effect of an improvement of the interlayer cohesiveness induced by the increase in the electrostatic charge of the slab with the iron content. The Ni–Ni distance, represented by the *a*-lattice parameter of the hexagonal cell, is 3.05 Å for NiFe15 and 3.00 Å for NiFe20. This variation is caused by the presence of the trivalent cation substituted for Nickel. As the ionic radius of Fe^3+^ is smaller than that of Ni^2+^ (*r*_*i*_ = 0.64 Å and *r*_*i*_ = 0.70 Å respectively), the Ni–Ni distance decreases with the iron content.^42^

**Table 1:** XRD data of the as-prepared and aged α-Ni_1−*x*_Fe_*x*_(OH)_2_ samples. *The reflections of the interstratified sample cannot be indexed to the d003 and d006 distance of the α-Ni(OH)2

Material (as prepared)	*Hkl*	*d*_obs_ (Å)	Cell parameter	Crystal size (nm)
NiFe7	E.P. (003*)	15.0	Interstratified phase	3.5
		6.88		
	(006*)	4.39		
NiFe15	(003)	8.64	*a* = 3.05 Å *c* = 25.92 Å	2.2
	(006)	4.18		
NiFe20	(003)	8.25	*a* = 3.00 Å *c* = 24.77 Å	1.7
	(006)	4.15		
Material (Aged)				
NiFe7	(003*)	5.58	Interstratified phase	—
	(006*)	4.34		
NiFe15	(003*)	6.07	Interstratified phase	3.8
	(006*)	4.13		
NiFe20	(003)	7.70	*a* = 3.07 Å *c* = 23.2 Å	5.7
	(006)	3.94		

The material NiFe7 (α-Ni_1−*x*_Fe_*x*_(OH)_2_ with *x* = 0.07) shows a peculiar X-ray diffractogram. Similarly, in the α-phase, the two reflections (003) and (006), that represent *C*_Hex/3_ and *C*_Hex/6_, are observed below 2*θ* = 30°. However, unlike for NiFe15 and NiFe20, their positions are not submultiples of one another (6.88 Å and 4.39 Å instead of 8.25 Å and 4.15 Å for material NiFe20) indicating that they cannot be indexed as the (003) and (006) reflections of an α-phase. Therefore, we will refer to these reflections as (003*) and (006*). This effect has already been observed by L. Guerlou-Demourgu *et al.*^43^ for a low trivalent cations concentration (<20 mol% in their case). It is typical of an interstratified structure where α and β-Ni(OH)_2_ domains coexist within a single crystallite. Estimation of the *β*/(*β* + *α*) ratio has been proposed in the literature by comparison of the experimental XRD patterns with the simulated XRD spectra.^43,44^ It has been found that for extreme values of the *β*/(*β* + *α*) ratio, the XRD patterns are similar to those of the corresponding pure phase. The general appearance of the NiFe7 spectra corresponds better to an intermediate scenario with *β*/(*β* + *α*) close to 0.5. The interstratified material obtained by L. Guerlou-Demourgues *et al.*^43^ for *x* = 0.1 showed a similar XRD pattern to sample NiFe7 with a first peak at 7.0 Å and a second one at 4.1 Å. By comparing the XRD pattern with simulated spectra they concluded that the ratio *β*/(*β* + *α*) in their sample is 0.55. In addition to these pseudo (003*) and (006*) reflections, the diffractogram of NiFe7 presents another particularity with an additional reflection at low angle (2*θ* = 6.85°, *d* = 15 Å) which could be attributed to an extra periodicity E.P. (this extra reflection is discussed in the ESI†). To conclude, a concentration >10% is then necessary to obtain a pure α-phase after the alkali precipitation synthesis.

Upon ageing in 6 M KOH, the various doped nickel hydroxides show sharper reflections suggesting an increased degree of crystallinity and an increase in the crystal size (Fig. 2). The crystal sizes before and after ageing are displayed in Table 1. Only material NiFe20 shows a pure α phase after 1 month of ageing, although the interlayer distance experienced a decrease from 8.25 to 7.70 Å. This may be due to an exchange of SO_4_^2−^ by CO_3_^2−^ upon ageing in KOH explained by the stronger affinity of carbonate with the LDH layers than other anions (*cf.* discussion in the ESI†).^45,46^

The diffractogram of aged NiFe7 still shows an interstratified behaviour with a (003*) reflection now shifted to a higher 2*θ* angle (*d* = 5.58 Å instead of 6.88 Å before ageing). This can be interpreted as an increase of the β-phase proportion within the interstratified structure. The positions of the peaks (003*) and (006*) are quite similar to those obtained by Rajamathi *et al.*^44^ with their interstratified Ni(OH)_2_ obtained by alkali precipitation which shows a first peak at 5.6 Å and a second peak at 4.2 Å. They conclude from DIFFax simulation that this material contains 60% of beta phase. The material NiFe15, which was showing pure α phase before ageing, also shows an interstratified structure now, with the shift of the (003) reflection to a higher 2*θ* angle (6.07 Å instead of 8.64 Å before ageing). The diffractogram appears indeed quite similar to that of NiFe7 before ageing.

To conclude, below a 20% iron concentration, the amount of intercalated anions balancing the excess of Fe^3+^ positive charge is not enough to uniformly fill the interlayer slab, leading to a segregation effect responsible for the interstratified material formation.^43^

The number of water molecules intercalated in the nickel hydroxide plays an important role in the crystal structure and electrochemical properties. TGA is used to determine the amount of water present in the samples. The TG and DTA curves of the samples are shown in the ESI,† Fig. S-3, and compared to those of the conventional β-Ni(OH)_2_. The content of adsorbed and intercalated water is then estimated at 18 wt% for all the doped samples and 8 wt% for conventional β-Ni(OH)_2_, Ni-B, which only contains adsorbed water. The amount of nickel in the samples (wt%) was determined by ICP and used later for the determination of the number of electrons exchanged per nickel atom (*cf.* section Material cost). The amount of nickel in the samples (wt%) as well as the Fe/(Ni + Fe) molar ratio determined by ICP are displayed in Table 2. The ICP results confirm the iron doping of the samples at 7, 13 and 18% which is close to the expected value (7, 15, and 20%). A chemical formula of the different samples is proposed and discussed in the ESI.†

**Table 2:** Chemical composition of the iron-doped samples determined using ICP analysis

Sample	Ni (wt%)	Fe (wt%)	Fe/(Ni + Fe) (%)
Ni-B	56.5	0.0	0.00
NiFe7	37.0	2.6	6.9
NiFe15	34.0	4.9	13.1
NiFe20	33.3	7.0	18.1

### Material cost

Considering the targeted application (GSESS), the electrochemical study focuses on characterising the performances of the material related to its cost per storage capacity unit, high-rate performance, energy efficiency and durability.

The parameter considered in this study to characterise the capacity of the material is the number of electrons exchanged per atom of nickel (NEE) rather than the specific capacity (milliampere-hour per gram of compound). Although the latter is conventionally used in the battery literature, as it is related to energy density, in the stationary storage considered here, the Ni content and therefore the material cost are of relatively higher importance.

**Fig. 3:**
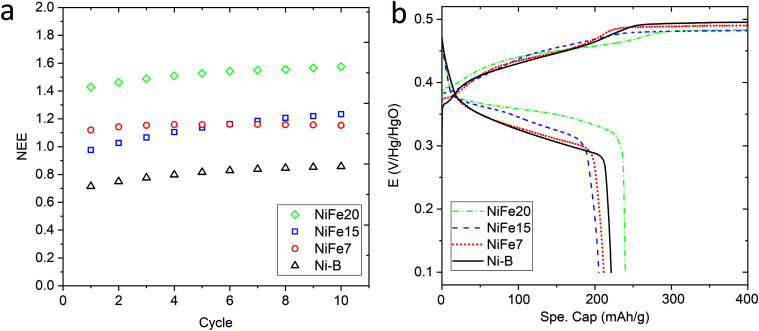
(a) Evolution of the NEE per Ni atom through the 10 activation cycles at 0.2C performed on NiFe20 (green diamond), NiFe15 (blue square), and NiFe7 (red circles), compared to β-Ni(OH)_2_ Ni-B (black triangle). (b) Charge and discharge curves as a function of the specific capacity (mA h g^−1^ of compound) for the 10th activation cycle C-rate = 0.2C.

Fig. 3a shows the evolution of the capacity along the 10 activation cycles at 0.2C for the three iron-doped samples compared to that of pure β-Ni(OH)_2_. As expected all the NiFe-LDH materials allow a higher number of electrons to be exchanged per nickel atom (between 1.15 and 1.57e^−^/Ni) than Ni-B that shows 0.86e^−^/Ni. From the doped samples, the NiFe20 material shows a much better performance than NiFe7 and NiFe15 which reach 1.15 and 1.23e^−^/Ni, respectively, at the end of activation. The electrochemical performance of the LDH materials can be correlated to their crystal structure. The interstratification of the alpha and beta phase layers in the crystal structure of samples NiFe7 and NiFe15 explains the lower capacity reached by these materials (*cf.* Material characterisation section). Indeed, only the alpha phase contains tetravalent nickel atoms allowing a higher number of electrons to be exchanged (NEE). In the interstratified material, the average oxidation state of nickel is then decreased by the presence of the beta phase layers. This interstratified structure is already observed before ageing for NiFe7. In the case of NiFe15, the transformation might occur during the preparation steps preceding the activation cycles (soaking of the electrodes and 1st long cycle) as well as during cycling. An assumption was made in the Material characterisation section that the ratio of the α/β phases was higher in NiFe15 after the ageing test than in the NiFe7 sample. This is confirmed by the higher capacity reached by NiFe15. The material NiFe20, which still showed a pure alpha phase after ageing in KOH, also gave the best results with 1.57e^−^/Ni.

When comparing with the literature, the material NiFe20 appears more performing than the 20%Fe doped Ni(OH)_2_ proposed by L. Demourgues-Guerlou *et al.*^47^ that shows a number of electron exchanged by metallic atoms (NEE/(Ni + Fe)) of 1.15e^−^ for the first cycles before it decreases. In comparison, NiFe20 gives a NEE/(Ni + Fe) of 1.29e^−^ at the end of the activation cycles. Also, all the doped materials studied in the present paper show a better capacity than the Zn doped Ni(OH)_2_ studied by Dixit *et al.*^28^ which shows a capacity of 500 mA h g^−1^(Zn) while NiFe7, NiFe15, and NiFe20 give, respectively, 574 mA h g^−1^(Ni), 603 mA h g^−1^(Ni) and 724 mA h g^−1^. On the other hand, the cobalt doped Ni(OH)_2_ discussed by R. Oesten *et al.*^48^ gives a slightly better capacity with a NEE/(Ni + Co) a bit higher than 1.3e^−^ compared to 1.29e^−^ for the NiFe20 samples. To conclude, sample NiFe20 gives a capacity relatively close to the highest number of exchanged electrons (NEE) reported in the literature which is 1.7e^−^/Ni^24^ but, more importantly, this constitutes an increase by a factor of 1.8 per Ni atom compared to the conventional β-Ni(OH)_2_, and without any use of cobalt. The amount of nickel in the hydroxide material, is then almost halved for a similar capacity, which is a large reduction in the required nickel resources (and cost).

The analysis of the (dis)charge curves of the different materials, shown in Fig. 3b, brings more insight into the cycling process. The shape of the charge and discharge curves, for example, reveals the composition of the material. The NiFe15 (dis)charge curves confirm the presence of two different phases (alpha and beta) highlighted by two plateaus visible in charge and discharge around the 100 mA h g^−1^ position. This effect is less visible for NiFe7 suggesting that the transformation from alpha to beta is almost complete for this sample.

Another important aspect is the charge and discharge potentials which appear to be significantly influenced by iron doping; a gradual increase of the potentials with iron concentration in the material is observed. The half-discharge potential (*Vd*_1/2_*vs.* Hg/HgO) of the different samples increases in this order:
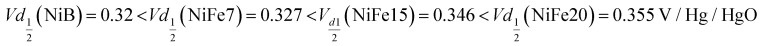


Although the effect of iron concentration is more remarkable during the discharge process, the same tendency is noticeable for the charge curve as revealed by the half charge potentials of the different samples:
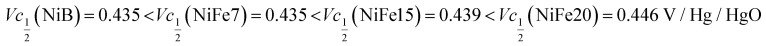


This shift towards higher potentials is also described by Demourgues-Guerlou *et al.*^30^ who report an increase of the oxidising character of γ-NiOOH with Fe substitution due to the presence of tetravalent iron in the charged phase. To gain more insights into this question, the equilibrium potentials at half state of charge (SOC = 1/2) have been determined by GITT measurements. The equilibrium potential of the Ni(OH)_2_/NiOOH redox couple is known to show a hysteresis behaviour, with the equilibrium potential *versus* SOC being higher when measured during the charge than during the discharge.^49,50^ This hysteresis behaviour could be related to a structural change induced by the intercalation (during discharge) and removal (during charge) of the proton in the Ni(OH)_2_ structure causing a lattice expansion and contraction.^49^ Thus, two equilibrium potentials are determined from the GITT measurement: 
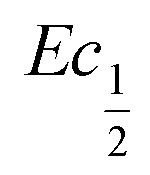
 and 
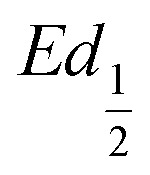
 obtained at SOC = 1/2 during the charge and discharge part of the GITT curve, respectively. Results are shown in Fig. S-4 (ESI†). The results reveal that there is indeed an increase in the equilibrium potential with the iron concentration in Ni(OH)_2_, reflecting a higher oxidation state.

The difference 
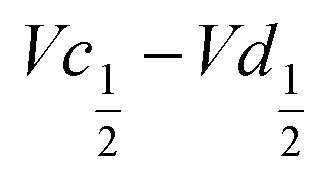
 also decreases with the iron content. Nevertheless, the difference in equilibrium potentials 
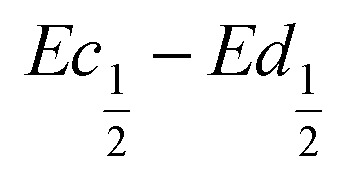
 is similar for all the samples (about 0.055 V), which proves that the kinetic charge rate dependent overpotentials are decreasing with the iron doping. This indicates a superior charge transport within the NiFe-LDH samples. This is confirmed by the EIS measurements. Fig. S-5 (ESI†) shows the Nyquist plots for each sample, they display a depressed semi-circle resulting from charge transfer resistance and a slope related to Warburg impedance in the low frequency region. The semi-circles are smaller for most of the Fe concentrated NiFe-LDH materials indicating an increased charge transport with Fe doping. The impact of the doping on the overpotential reduction can find different explanations. First, the ionic pathways with the stabilised large interlayer gap of the alpha structure will be enhanced, reducing ionic resistances; and second, doping enhances the electronic conductivity of the material.

Concerning electrolysis, the overpotential for OER is visible beyond 300 mA h g^−1^ charge inserted, shown in Fig. 3b. The potential is also impacted by the catalytic behaviour of iron doping and decreases with the concentration of doping:
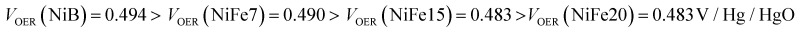


The OER potentials reached by all samples are above their equilibrium potential (0.28 V/Hg/HgO) but below the thermoneutral potential of OER (0.601 V/Hg/HgO), which is possible because of the external heat coming from the environment. Calculation for thermoneutral and equilibrium potential of OER is detailed in the ESI.†

Sample Ni-B appears to offer more capacity than NiFe7 and NiFe15 when considering the specific capacity in milliamp-hour per gram of compound, while the estimation in NEE/Ni presented earlier gives a different tendency. This is explained by the higher Ni content per gram of the compound in the Ni-B material which does not contain Fe doping and has no water intercalated, which compensates for the lower NEE per Ni. However, for the sample NiFe20 both a higher specific capacity and a much higher capacity per Ni amount than the β-Ni(OH)_2_ are reached. This indicates that despite the reduced Ni amount in the compound and the enhanced OER leading to a lower faradaic efficiency of the sample, the high number of electrons exchanged per nickel by NiFe20 enables the battery gravimetric energy density to be increased as well. Nevertheless, it has to be noted that some β-Ni(OH)_2_ materials found in the literature have higher specific capacity than the Ni-B sample discussed in the present paper. For instance, the Co and Zn doped β-Ni(OH)_2_ proposed by X. Yue *et al.*^51^ gives specific capacity in the range of 250–300 mA h g^−1^. Compared to these materials, the sample NiFe20 gives better NEE but lower specific capacity per mass of active material.

### High-rate performances and energy loss

To be suitable for grid-stabilisation, an energy storage device must be able to charge and discharge at sufficiently high rates. Typically, electricity storage systems are designed to reach 4 hours of storage duration.^52^ Besides, in ref. 53 one can observe that renewable solar and wind based energy also follows diurnal behaviour with about 4 h electricity overproduction periods. Thus, an advantageous and realistic use of a battolyser on a daily basis would consist in applying a charge rate of 1C to fully charge the battery in 1 hour for short-term storage (to provide electricity at night) and producing hydrogen for the next 3 hours for long term storage and as chemical feedstock. Note that for these two applications, the round trip efficiency for H_2_ back to electricity (≈35%) may justify generating 75% of the charge time hydrogen to store the same amount of electricity as for the battery. Thus more hydrogen needs to be stored when aiming to generate electricity later in the year, and the feedstock application ads to that. Charge rates of 1C, maintained for 4 hours, are therefore important to target. Discharge rates of 4 hours are in general sufficient.

Few studies have investigated the high rate performance of α-Ni(OH)_2_ but the charge rate applied usually do not exceed 5C.^21,23,54,55^ Besides, it is often unclear whether the electrode theoretical capacity and the charge rate are calculated based on the theoretical specific capacity of β-Ni(OH)_2_ (289 mA h g^−1^) or of α-Ni(OH)_2_ (490 mA h g^−1^) and if the weight considered is the total sample mass (including the doping and the water content) or only the mass of the active species present in the sample. For these reasons, comparison with the literature is difficult. In this study the impact of the charging rate, C-rate, on the materials capacity has been evaluated by increasing the C-rate from 0.1C to 4C considering a theoretical specific capacity of 490 mA h g^−1^ and the total mass of the sample (doping and water included). Therefore, 0.1C corresponds to 49 mA g^−1^ (≈0.1 mA cm^−2^) and 4C to almost 2 A g^−1^ (≈4 mA cm^−2^).

**Fig. 4:**
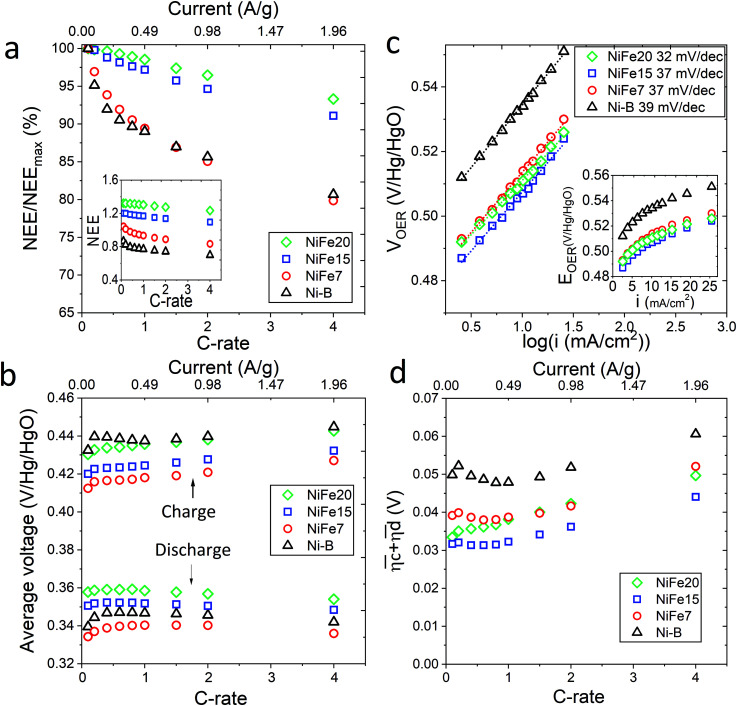
High rate performances of β-Ni(OH)_2_ and LDH-Fe-Ni(OH)_2_ materials. (a) Evolution of the discharge capacity with the C-rate represented as the ratio of the discharge capacity to the discharge capacity at a C-rate of 0.1C and, in the inset, as NEE, (b) average voltage of the (dis)charge curve for the different C-rates, (c) iR corrected OER Tafel plots and, in the inset, evolution of *V*_OER_ with current density, and (d) sum of the kinetic overpotentials for different C-rates.

The loss of discharged capacity induced by the current increase is represented in Fig. 4a with the NEE normalised by the value of NEE at 0.1C *versus* the C-rate. This reveals that iron doping has a significant impact on the response of the material to a current increase. Indeed, the discharge capacity reduction induced by the current increase is less for the doped samples and is gradually reduced with the increase of Fe concentration in the material. While the Ni-B material loses 19% of its discharged capacity with the C-rate increasing from 0.1 to 4C, only 7% is lost by NiFe20. This can be explained by the better ionic conduction of the protons through the material allowed by the high interlayer distance and water content of the alpha phase, and also by an improvement of the electronic conductivity induced by iron doping. When compared to the literature, it appears that some alpha-Ni(OH)_2_ materials showing higher specific capacity at 0.2C than NiFe20 do not resist as well to an increase of the charge rate. For instance, the Ca^2+^/PO_4_^3−^ doped alpha/beta Ni(OH)_2_ studied by C. Miao *et al.*^56^ shows a specific capacity of 271 mA h g^−1^ at 0.2C but 238 mA h g^−1^ at only 0.5C, which constitute a decrease of 12.5%. Similarly, the Al-doped alpha nickel hydroxide proposed by X. Wu *et al.*^23^ gives a high specific capacity of 301 at 0.2C but only 185 mA h g^−1^ at 5C, a loss of 39%. In comparison, the capacity loss observed for the NiFe20 material are surprisingly small. The charge rate of 1C desired for the integrated battery–electrolyser operation is then perfectly realistic since, for the sample NiFe20, it implies only 1% loss compared to its low current discharged capacity (at 0.2C).

Before concluding on the high rate performances of the materials, other criteria have to be taken into account such as the overpotentials reached during the charge and discharge of the materials to ensure that an increase of the C-rate is not detrimental for the energy efficiency of the storage device.

The energy loss related to the use of a nickel electrode within a hybrid battery electrolyser device can be decomposed into the battery losses, related to the nickel electrode (dis)charge irreversibility, and the OER loss (*cf.* Fig. S-6 and eqn (S-9) in ESI†).

The nickel electrode battery losses (in Joule) can be expressed as follows:1
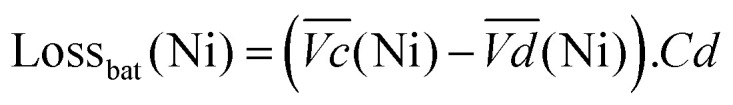
where 
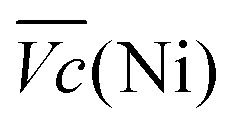
 and 
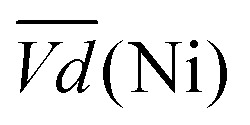
 are the average potential of the charge and discharge processes (*cf.* ESI,† for eqn (S-12) and (S-13)), and *C*_*d*_ the discharge capacity. Thus, the contribution of the nickel electrode battery function to the hybrid battery energy efficiency losses can be estimated by comparing Loss_bat_(Ni) to the energy inserted to the full cell, for the same operating conditions (a more detailed calculation is given in the ESI†).2
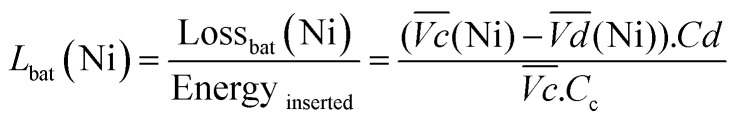
where *C*_*c*_ = 2*C*_*d*_ is the chosen charge inserted (so half of the charge converted to H_2_), and 
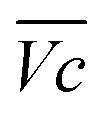
 is the average voltage of the full cell charge estimated at 1.6 V.

Thus, the difference between 
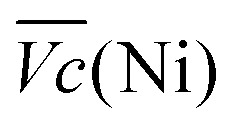
 and 
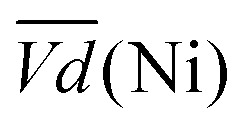
 is an interesting criterion to characterise the energy loss. Fig. 4b shows the impact of C-rate on 
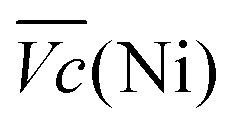
 and 
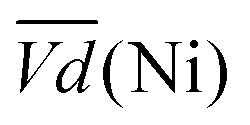
 Ni-B shows the highest 
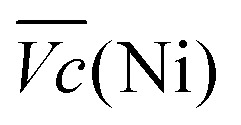
 for all C-rates and the second lowest 
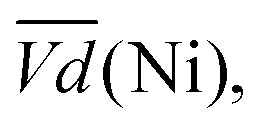
 which results then in a higher energy loss than the NiFe-LDH samples. The energy efficiency loss *L*_bat_(Ni) related to the nickel electrode (dis)charge processes (calculated according to eqn (2) with *C*_*C*_ = 2*C*_*d*_) corresponds to 2.9% for Ni-B and 2.3% for NiFe20 at 0.1C, when assuming a full cell charging with a 
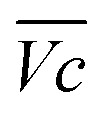
 of 1.6 V. For all C-rates the use of NiFe20 instead of Ni-B leads to a reduction of the energy loss of −0.4 to −0.7%, this represents a reduction by 12 to 24% compared to the Ni-B loss.

For both NiFe20 and Ni-B, Loss_bat_(Ni) at 4C is slightly higher than at 1C (*cf.* Table S-2 in ESI†). This corresponds to an increase of the energy efficiency loss *L*_bat_(Ni) from 2.3% to 2.8% for NiFe20 and from 2.9 to 3.3% for Ni-B, according to eqn (2).

Remarkably, the 
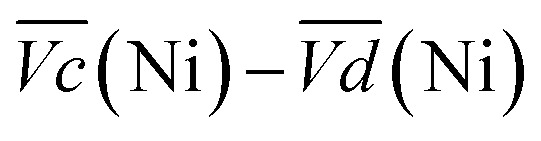
 difference is not only composed of overpotentials related to kinetic effects but also of overpotentials related to the hysteretic effect of the equilibrium potentials (*cf.* Fig. S-6 in the ESI†). Thus, for a better insight into the kinetic and hysteresis contributions to the energy loss, the sum of the kinetic discharge and charge overpotentials is determined *via*eqn (3):3
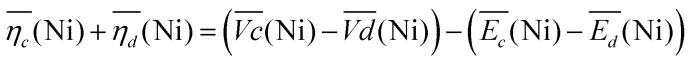
where 
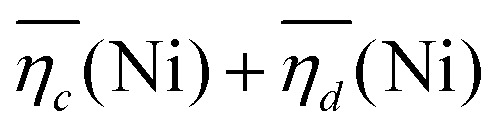
 is the sum of overpotentials averaged over the charge and discharge processes, 
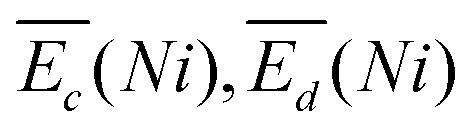
, the equilibrium potentials averaged over the charge and the discharge of the samples (*cf.* ESI,† eqn (S-19) and (S-20)) and determined by GITT (Fig. S-4 in ESI†).

The sum of the overpotentials 
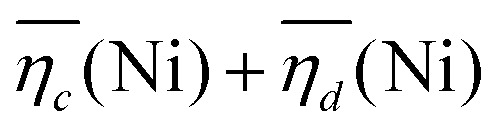
 is represented in Fig. 4d as a function of the C-rate. Thus, for all C-rates the overpotentials are higher for β-Ni(OH)_2_ than for the doped samples. Thus, the kinetic energy loss *L*_kinetic_(Ni) for materials Ni-B and NiFe20 at 4C represents 1.9% and 1.5% of energy efficiency loss, respectively, for a full cell charging at 
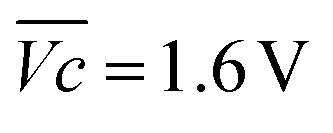
 and *C*_c_ = 2*C*_d_ according to eqn (4). This decrease of the kinetic overpotential induced by doping can be explained by a better ionic and electronic pathway as explained earlier and will allow a reduction of the energy loss.4
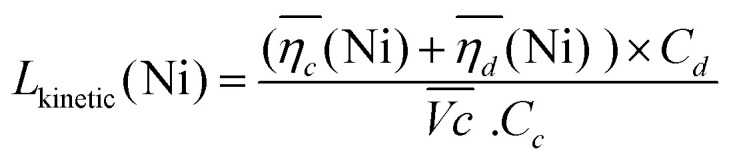
5
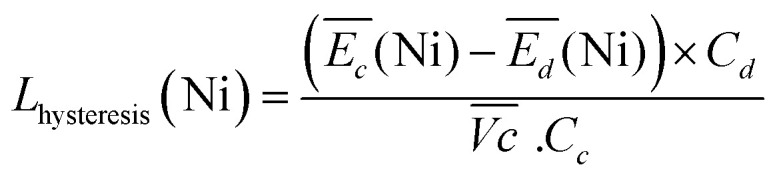


For the same charge rate, the hysteresis contribution to the energy efficiency losses corresponds to 1.4% and 1.2% for Ni-B and NiFe20, respectively, according to eqn (5).

Unlike the kinetic loss, the hysteresis loss appears intrinsic to the structural changes of nickel hydroxide material during (dis)charge and is therefore unavoidable. It is also worth noticing that at a low C-rate (0.1C) this hysteresis loss is higher than the kinetic loss. For Ni-B it represents an energy efficiency loss of 1.6% while the kinetic loss is 1.4%. The same tendency is observed for the NiFe-LDH samples.

For a conventional NiFe battery function, a high OER potential is necessary to have a good energy efficiency because it implies a higher faradaic efficiency of the cycling process. In contrast, for the hybrid battery-electrolyser function proposed here, the faradaic efficiency is not affected by the water splitting reaction because the hydrogen and oxygen are useful products. In this case, a decrease of the OER overpotential is even desirable to allow an improvement of the energy efficiency. The catalytic activity of the nickel hydroxide materials towards OER is characterised by chronopotentiometry with current densities ranging from 0.6 to 25 mA cm^−2^. The advantage of characterising the OER catalytic activity by galvanostatic experiments instead of cyclic voltammetry is that, unlike with cyclic voltammetry, bubbles formed on the electrode can be removed before each point of the Tafel plot avoiding a reduction of the electrode active surface. Also, every point is obtained at a steady state. The results are displayed in Tafel plots in Fig. 4c and confirm that the doped nickel hydroxides outperform the pure nickel hydroxide in activity and kinetics. Indeed, both the overpotentials and the Tafel slope are lower for NiFe-LDH materials. Material NiFe20 shows a Tafel slope of 32 mV decade^−1^ and an overpotential of 205 mV at 10 mA cm^−2^, while the slope is of 39 mV decade^−1^ and the overpotential at 10 mA cm^−2^ of 230 mV for Ni-B.

The better catalytic behaviour of the NiFe-LDH material compared to that of the pure beta phase can be explained by the presence of iron in the material, which makes it a well-known OER catalysts. Recent studies have investigated the reason behind the enhanced activity of the NiFe-LDH compound focusing on defining the active sites and the role of Fe on the NiFe-LDH OER activity.^57–59^ Several authors^57,58^ agree that the actives sites in Ni_(1−*x*)_Fe_*x*_OOH are the Fe cations. Friebel *et al.*^57^ demonstrated that Fe^3+^ cations occupy octahedral sites in the NiFeOOH material with unusually short Fe–O bond distances due to edge-sharing with the surrounding NiO_6_ octahedra. DFT calculation showed that this structural motif results in near optimal adsorption energies of OER intermediates at the Fe sites leading to an increase in OER catalytic activity. Nevertheless, the superior catalytic activity of Fe-α-Ni(OH)_2_ compared to β-Ni(OH)_2_ may not only be attributed to the Fe cations. Y. Xu *et al.*^60^ have demonstrated that the crystallinity of the material also plays a role in the OER. They showed that a small crystal size, a low crystallinity and a large interlayer distance are favourable for an efficient OER due to more exposed active sites, lower charges transfer resistance, and better exchange ability with OH^−^. Therefore the good OER performance of the NiFe-LDH materials can also be attributed to their low crystallinity (Fig. 2) and large interlayer distance (Table 1).

NiFe-LDH is widely investigated for the water splitting application due to its activity for OER.^33,36,46,61–65^ The overpotentials and Tafel slope obtained in this study are comparable to those found in the NiFe-LDH literature.^61–63,66^ Oliver-Tolentino *et al.*^63^ obtained NiFe-LDH materials exhibiting Tafel slope of 36–37 mV dec^−1^ while C. Kuai *et al.*^61^ manage to reach 31 mV dec^−1^ with their ultrathin NiFe-LDH and an overpotential of 210 mV at 10 mA cm^−2^. Also, NiFe-LDH materials tested in the present study outperformed the well-known Ir/C catalyst which exhibits a Tafel slope of 40 mV dec^−1^.^66^ Due to these excellent catalytic properties NiFe-LDH can be used for efficient water splitting once the Ni/Fe hybrid battery is fully charged.

The energy efficiency loss related to the OER overpotential can be estimated from the difference between the OER plateau of the charge curves and the thermoneutral potential for OER of different samples (0.601 V *vs.* Hg/HgO *cf.* calculations in ESI†):6
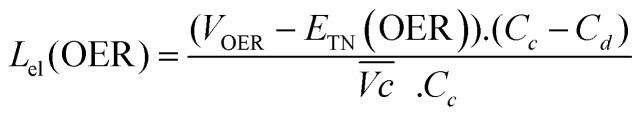
where *E*_TN_(OER) is the thermoneutral potential of OER, and *V*_OER_ is the potential of the OER plateau.

As shown in Fig. 4c, *V*_OER_ is lower than E_TN_(OER) for all samples at all C-rates applied. This can be explained by external heat coming from the environment and implies a negative value for the energy efficiency losses *L*_*el*_(OER). For a full cell charging with 
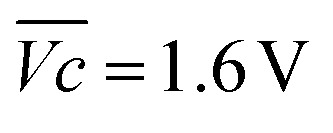
 at 4C, the OER energy efficiency losses are estimated at −3.2% for NiFe20 instead of −2.5% for Ni-B inducing a gain in energy efficiency of +0.7% for the doped sample. Combined with the gain in battery energy efficiency, the use of NiFe20 constitutes an increase in total energy efficiency of +1.1 to +1.4%; since the typical full cell efficiency is 80–90%,^1^ this constitutes a reduction of the overall full cell losses by 7–14%.

### Stability

**Fig. 5:**
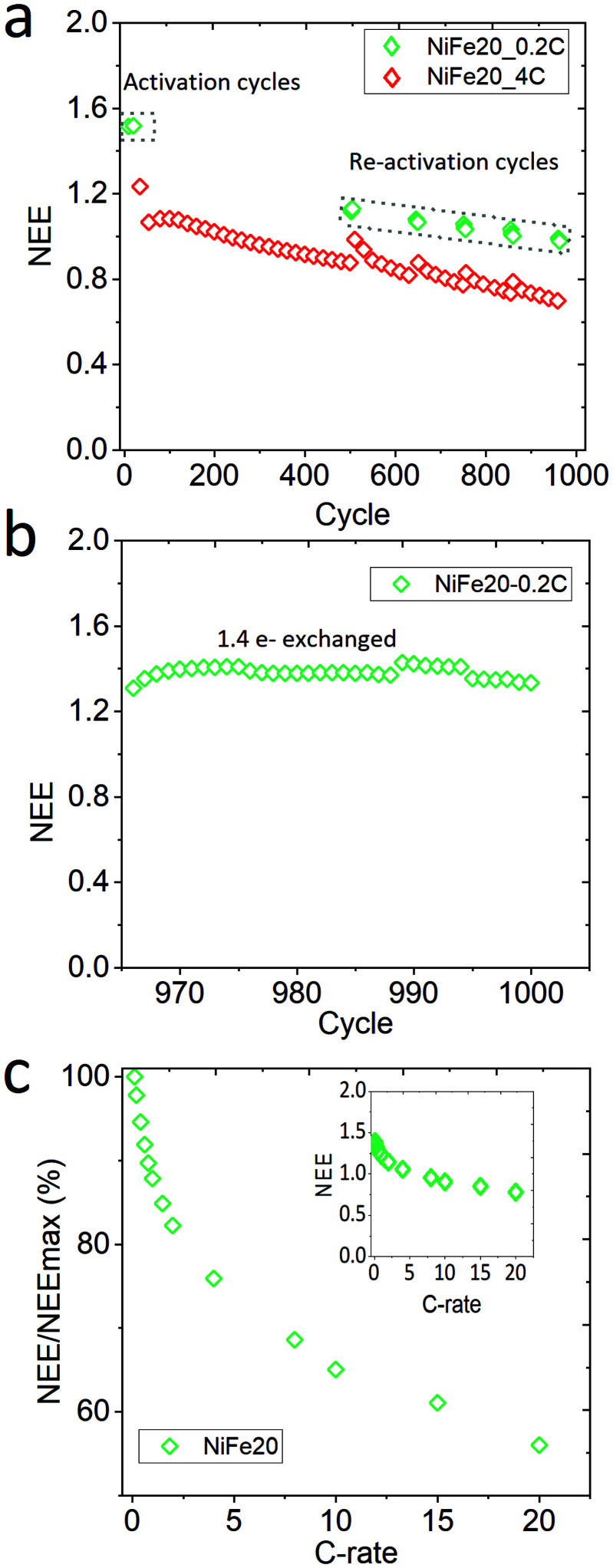
Characterisation of NiFe20 stability: (a) long-term stability test, (b) after the long-term stability test the electrode is repressed and the capacity of NiFe20 goes back to 1.4e^−^ exchanged, and (c) high rate performance of the repressed electrode after the long-term stability test represented by NEE normalised by NEE at 0.1C.

Sample NiFe20, which gives the best capacity performance, has been exposed to a life cycle experiment to characterise its stability over the cycling. After the activation and C-rate experiments the electrode has performed 960 cycles at 4C charge, overcharge, and discharge. Mid-way in the life cycle experiments, 6 reactivation cycles at 0.2C were performed for every 100 cycles. Finally, at the end of the 960 cycles the electrode was re-pressed to its initial thickness to reconnect the material with the current collector, and the electrode was cycled again at 0.2C. In total the electrode performed 1000 cycles. The whole history of the electrode is represented as NEE in Fig. 5.

A constant decrease of the capacity is observable along the 960 cycles in Fig. 5a. The reactivation cycles performed during the second step of the experiment highlight that it is possible to regain some extra capacity by (dis-)charging the material more slowly. This suggests that some part of the material cannot be reached at such a high (dis-)charge rate due to a weakening of the conductivity paths within the electrode. However, the capacity reached during the reactivation cycles also shows a clear decrease over time. In order to determine if the decrease of the capacity is due to the ageing of the material or to a loss of electronic contact, the electrode is re-pressed and reactivated at 0.2C (Fig. 5b). This results in a net increase of the capacity with a NEE going up to 1.41e^−^/Ni. The decrease of capacity observed over the long-term stability test can thus be explained by a loss of contact between the Ni(OH)_2_ particles and the current collector, probably induced by the oxygen bubbles generated during the repetitive overcharges. Nevertheless, it can be concluded that the NiFe20 material itself is able to withstand a large number of cycles. After 1000 cycles its capacity corresponds to 90% of the capacity obtained after the first 10 activation cycles (NEE = 1.57 Fig. 3). A comparison with the literature is difficult because the stability of α-Ni(OH)_2_ is not often studied over more than 200 cycles. Nevertheless, the loss of capacity reported over the first 100 cycles is often already 10 to 40%.^23,25,30,56,67,68^

On top of the capacity stability, the OER catalytic stability has also been observed. Fig. S-7 in ESI,† shows the OER catalytic stability of the NiFe20 material over almost 200 hours of charge at 4C (∼4 mA cm^−2^) and shows that the OER overpotential remains stable around 200 mV. In addition, the sample NiFe20 still shows a good high rate performance even after 1000 cycles (Fig. 5c). It is able to withstand a C-rate as high as 20C for both charge and discharge, still giving an excellent high number of electrons exchanged (0.8e^−^/Ni, or in other terms, 46% SOC can be reached in 3 minutes).

The stability of the alpha phase within NiFe20 is also confirmed by XRD and XPS analyses. The XRD diffractogram of the aged electrode (Fig. S-9 in ESI†) highlights that the NiFe20 material is still essentially α-Ni(OH)_2_ after 1000 cycles. A very small peak corresponding to β-Ni(OH)_2_ is also observable and could explain the small decrease in capacity from 1.57e^−^ to 1.4e^−^ along the 1000 cycles. Nevertheless, the results indicate the high stability of the crystal structure. This is also beneficial for the mechanical stability of the electrode which, when a β-Ni(OH)_2_ material is used, suffers from the swelling of the material. The surface composition of the aged NiFe20 electrode is analysed by XPS and compared to that of a fresh electrode (Fig. S-10, ESI†). For both the fresh and aged electrodes, the high resolution Ni 2p spectra (Fig. S-10b, ESI†) can be fitted into 2 spin–orbit peaks Ni 2p_1/2_ and Ni 2p_3/2_ at ∼855.6 eV and 873.31 eV with two satellite peaks indicating that Ni is in the +2 oxidation state.^38,63^ Binding energies for Fe 2p_3/2_ and Fe 2p_1/2_ are positioned at ∼712 eV and ∼725 eV confirming the presence of +3 Fe.^69–71^ Again, the spectra of fresh and aged electrodes are quite similar (Fe 2p_3/2_ at 712.6 eV and 712.1 for the fresh and aged electrodes, respectively, and, Fe 2p_1/2_ at 725.2 eV and 725.1 eV) which confirms the stability of the material. Further stabilisation of the electrode capacity will require mitigation measures to fix the electrode morphology, such as enclosing the active material in volume fixed metallic pockets, as well as operating at elevated pressures to reduce the volume of the gas bubbles (*e.g.* 10 Bar operation reduces this volume tenfold). The first approach is common in Ni/Fe batteries and the second in alkaline electrolysis.

## Conclusions

Ni–Fe layered double hydroxides have been investigated for the first time for a hybrid battery-electrolyser application. Thus, battery properties, including storage capacity, rate performance, and cycling stability, as well as catalytic OER activity, have been characterised. The Fe doped materials appear beneficial from the following aspects:

– The stabilisation of the alpha/gamma phase couple that allows avoiding the swelling of the electrode and ensuring a better mechanical integrity through the charge, discharge and electrolysis processes.

– Increased capacity per nickel atom by 83% compared to the conventional beta phase positive electrode material.

– Enhanced ionic and electronic conductivity enabling the NiFe-LDH to be (dis-)charged at a high rate with a lower impact on the capacity (reduced by only 7% at 4C), and at reduced overall energy loss (reduced by 7 to 14%).

With these advancements, the NiFe-LDH can address the problems of Ni cost and energy efficiency, as well as stability aspects that are relevant for the implementation of the hybrid Ni/Fe battery-electrolyser concept in grid electricity storage and conversion.

## Experimental section

### Material preparation

Fe-substituted nickel hydroxide materials containing 7%, 15%, and 20% (NiFe7, NiFe15, and NiFe20), were prepared by a simple chemical co-precipitation method. The precipitation is carried out a constant high pH to avoid the formation of iron(iii) oxides/hydroxides. A solution of iron and nickel sulphate salts mixed in the appropriate ratio was slowly dropped into a 2 M NaOH solution under stirring. The pH-value of the mixture solution is controlled to be 13.2–13.4 during the whole synthesis. The precipitate was separated from the solution by centrifugation and washed with deionised water (this procedure was repeated twice). The precipitate was then dried in a vacuum oven at 50–60 °C until a constant weight was reached. The obtained materials were then ball-milled at 200 RPM for 12 min. For comparison purposes, pure β-Ni(OH)_2_ was synthesised following the same protocol. A schematic illustration of the synthesis procedure is proposed in Fig. S-2 (ESI†).

### Material characterisation

The phase structure of the as-prepared and aged samples was identified using a Bruker D8 Advance diffractometer with Co K_α_ source (*λ* = 1.78890 Å, 35 kV and 40 mA) and LynxEye position sensitive detector. The scan data were collected in a 2*θ* range of 5–95° with a step size of 0.06° and a counting time of 15 s. TG and DTA measurements were performed using a TGA2 from Mettler Toledo under air flow and in a temperature range of 30–800 °C with a step of 10 °C per minute. The metal content of the prepared samples was analysed using an ICP-OES, Spectro Arcos EOP. XPS spectra was obtained using a Thermo Scientific K-Alpha XPS system equipped with an Al K-Alpha X-ray source and a flood gun for charge compensation of the sample. Parameters used for the measurements were: spot size, 400 μm; pass energy, 50 eV; energy step size, 0.1 eV; dwell time, 50 ms; 10 scans in the vicinity of the Ni 2p, C 1s and O 1s orbital binding energy and 25 scans in the vicinity of Fe 2p orbital binding energy.

### Electrode preparation

Pasted nickel electrodes were prepared as follows: 50% of Ni(OH)_2_, 25% of carbon super P and 25% of graphite were ground together before adding a polyethersulfone (PES) solution to the mixture (7 wt% in NMP) until a homogeneous slurry was obtained. The slurry was then pasted into a nickel foam which was cut beforehand in a disk-shape of 1 cm diameter, and treated under ultrasound for 3 min in HCl (4 wt%) and 3 min in acetone in order to remove the oxide layer. After pouring the active material into the nickel foam, the electrodes were soaked in water to induce the precipitation of the polymer by a phase inversion process.^72^ The electrodes were then dried under vacuum at 50–60 °C and pressed to a thickness of 0.1 mm (1/5 of the initial thickness) to ensure a good electric contact between the foam and the active material. The morphology of the electrodes has been observed by SEM (SEM-JEOL6010LA). The SEM images of NiFe20 electrodes before and after 1000 cycles are shown in Fig. S-8, in the ESI.† Finally, the electrodes were wrapped into a nickel perforated tape. A blank electrode was prepared following the same protocol but without adding nickel hydroxide to the slurry.

### Electrochemical characterisation

The electrochemical tests were performed in 6 M KOH with a three-electrode cell, the working, the counter and the reference electrodes being, respectively, the Ni(OH)_2_ pasted electrodes, a nickel foil and a Hg/HgO (6 M KOH) reference electrode. The potential of the Hg/HgO reference electrode was estimated using: *E*(Hg/HgO) = 0.098 − (RT/*F*)ln[OH^−^] = 0.052 V/SHE. The pasted electrodes were soaked in the electrolyte (6 M KOH solution) for 10 hours before starting the electrochemical tests. The electrochemical performance including activation cycles, long-term cycling and high-rate acceptance tests were conducted using a Maccor 4000 battery cycling system. The theoretical capacity, for all samples, was calculated based on the theoretical specific capacity of pure α-Ni(OH)_2_ (490 mA h g^−1^) corresponding to 1.7e^−^ per Ni (maximum number of electrons exchanged that has been reported in the literature for a nickel hydroxide sample^24^). Knowing that the NiFe-LDH materials discussed here are not pure nickel hydroxides (they contain doping, water and anions), the expected capacity is actually lower. For all charge cycling experiments the charge inserted was 1.5 times the theoretical capacity to simulate both full charging and electrolysis in each cycle, and the (dis)charge rate was 0.2C unless mentioned otherwise. The discharge capacity values were corrected using the blank electrode discharge capacity corresponding to the formation and reduction of nickel oxide formed on the nickel substrate when cycling. Electrochemical impedance spectroscopy (EIS) measurements were carried out using a Parstat MC Multichannel potentiostat, at OCP on discharged electrodes from 10^5^ to 0.001 Hz with an AC potential amplitude of 5 mV. Tafel plots were obtained on the already charged materials by chronopotentiometry with current densities from 2.5 mA cm^−2^ to 25 mA cm^−2^. For this experiment, a rotating bar is placed below the working electrode to remove the generated bubbles. The *V*_OER_ potentials were corrected with IR compensation and the OER overpotential at 10 mA cm^−2^ is estimated using: *η*_OER_ = *V*_OER_ − 1.23 + 0.059.pH + 0.052.

## Conflicts of interest

FM is involved in Battolyser BV, an organisation that aims to scale up the hybrid battery-electrolyser concept for electricity storage and conversion applications.

## Supplementary Material

MA-002-D1MA00024A-s001
