# Supplementary material for: Nickel-iron layered double hydroxides for an improved Ni/Fe hybrid battery-electrolyser
Source: Mater Adv. 2021 May 25;2(15):5076–88. doi: 10.1039/d1ma00024a (PMC8328080; doi:10.1039/d1ma00024a)
Supplement: MA-002-D1MA00024A-s001 [file MA-002-D1MA00024A-s001.pdf]

Electronic Supplementary Material (ESI) for Materials Advances.
This journal is © The Royal Society of Chemistry 2021

ELECTRONIC SUPPLEMENTARY INFOMATION

Nickel-iron layered double hydroxides for improved Ni-Fe hybrid battery-electrolyser positive electrodes

A. Iranzo,[a] F.M. Mulder [a]

[a.] Materials for Energy Conversion and Storage (MECS), Department of Chemical Engineering, Delft University of Technology, Van der Maasweg 9, 2629HZ Delft, The Netherlands.

Ni-Fe hybrid battery-electrolyser operation principle

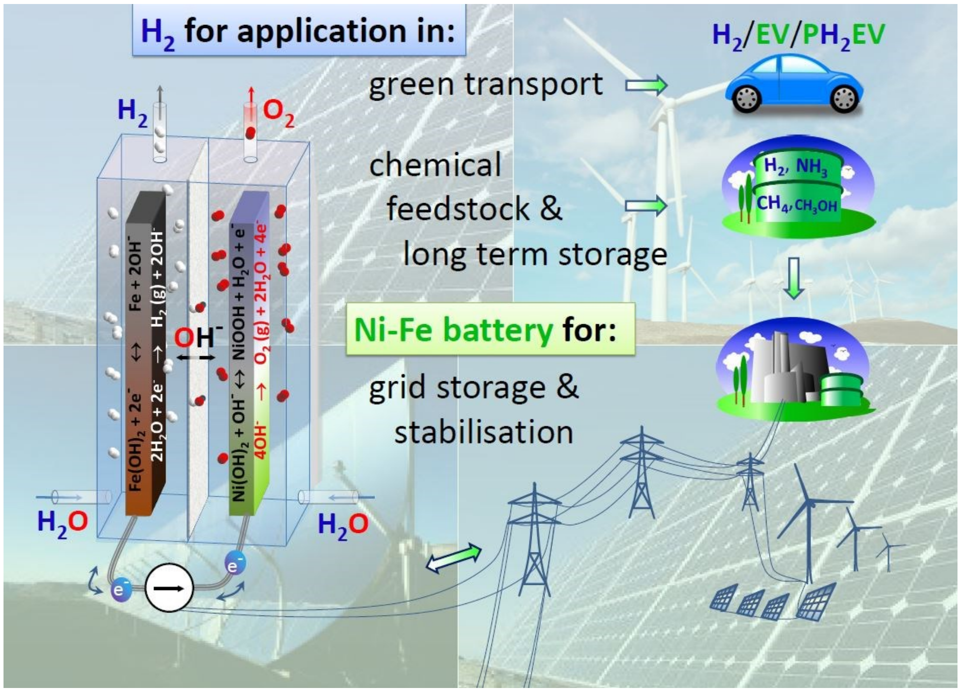

Figure S- 1: Operation principle of the Ni-Fe hybrid battery-electrolyser (battolyser). [1]

Figure S- 1 illustrates the Ni-Fe hybrid battery-electrolyser and the function it can provide in several potential application areas. The device has a negative electrode in which $Fe(OH)_2$ is reduced to Fe upon charge (-0.88 V relative to the standard hydrogen electrode (SHE)):

$$Fe(OH)_2 + 2e^- \xrightarrow{charge} Fe + 2OH^- \qquad \text{Eq-S 1}$$

When the positive electrode contains β-$Ni(OH)_2$, β-NiOOH is formed upon charge (+0.49 V vs. SHE):

$$Ni(OH)_2 + OH^- \xrightarrow{charge} NiOOH + H_2O + e^- \qquad \text{Eq-S 2}$$

For an electrode containing the α-phase $Ni_{(1-x)}Fe_x(OH)_2A_x$, the charge reaction may be described as follows assuming that only Ni changes its valence, while Fe remains Fe^{3+}, and the additional $+x$ positive charge in the lattice from the Fe_x is compensated by the anions with effective charge $-x$:

$$Ni_{(1-x)}Fe_x(OH)_2A_x + 1.5(1-x)OH^- \overset{charge}{\rightarrow} Ni_{(1-x)}Fe_x(OH)_{2x}A_x\,(OOH_{0.5})_{(1-x)} + 1.5(1-x)H_2O + 1.5(1-x)e^- \quad \text{Eq-S 3}$$

During the overcharge, the following reactions take place on the reduced iron and oxidised nickel hydroxide electrode respectively:

$$2H_2O + 2e^- \overset{overcharge}{\rightarrow} + H_2(g) + 2OH^- \quad \text{Eq-S 4}$$

$(0 - 0.059 \times pH\ vs. SHE)$

$$4OH^- \overset{overcharge}{\rightarrow} + O_2(g) + 2H_2O + 4e^- \quad \text{Eq-S 5}$$

$(1.23 - 0.059 \times pH\ vs. SHE)$

Material synthesis

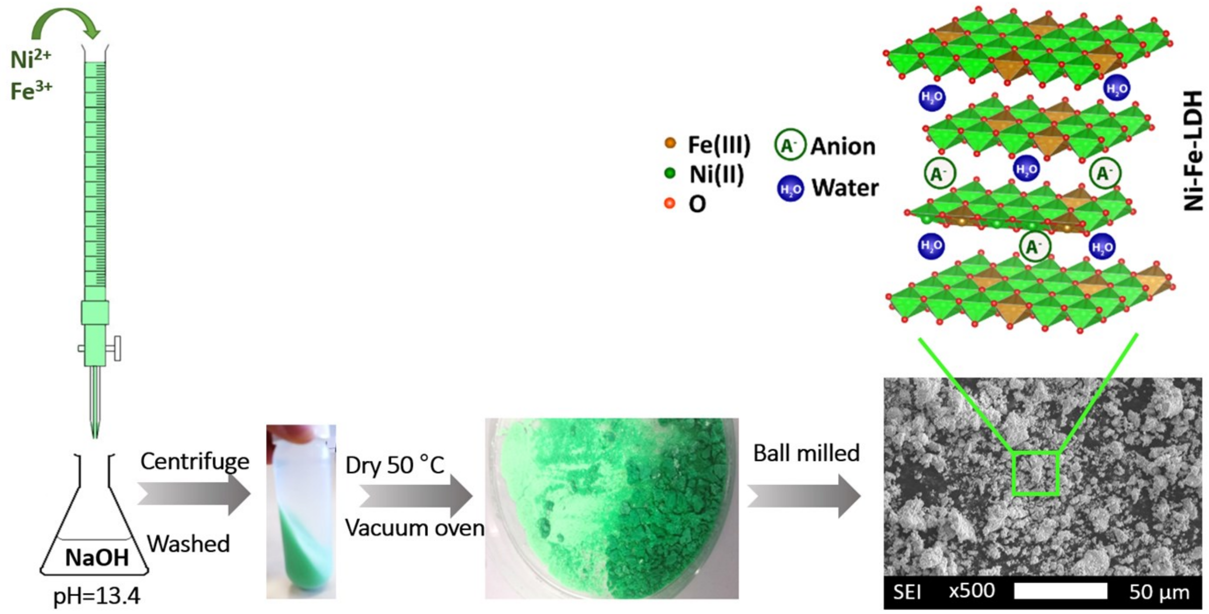

Figure S- 2: Schematic illustration of the synthesis protocol for the production of NiFe-LDH

Material characterisation

- **Crystal structure**

The extra peak observed at low angle in XRD spectra of the material NiFe7 (2θ=15°) reveals the presence of an extra periodicity (E.P) in the c-axes of the interstratified material which could correspond to the repetition of a sequence of one alpha inter slab distance (6.88 Å), and two beta interslab distances (4.39 Å), adding up and forming a [dα dβ dβ] reflection. To our knowledge, this periodicity has not been mentioned before and reveals a certain regularity and organisation in the interstratification of the alpha and beta phases.

- **Chemical formula**

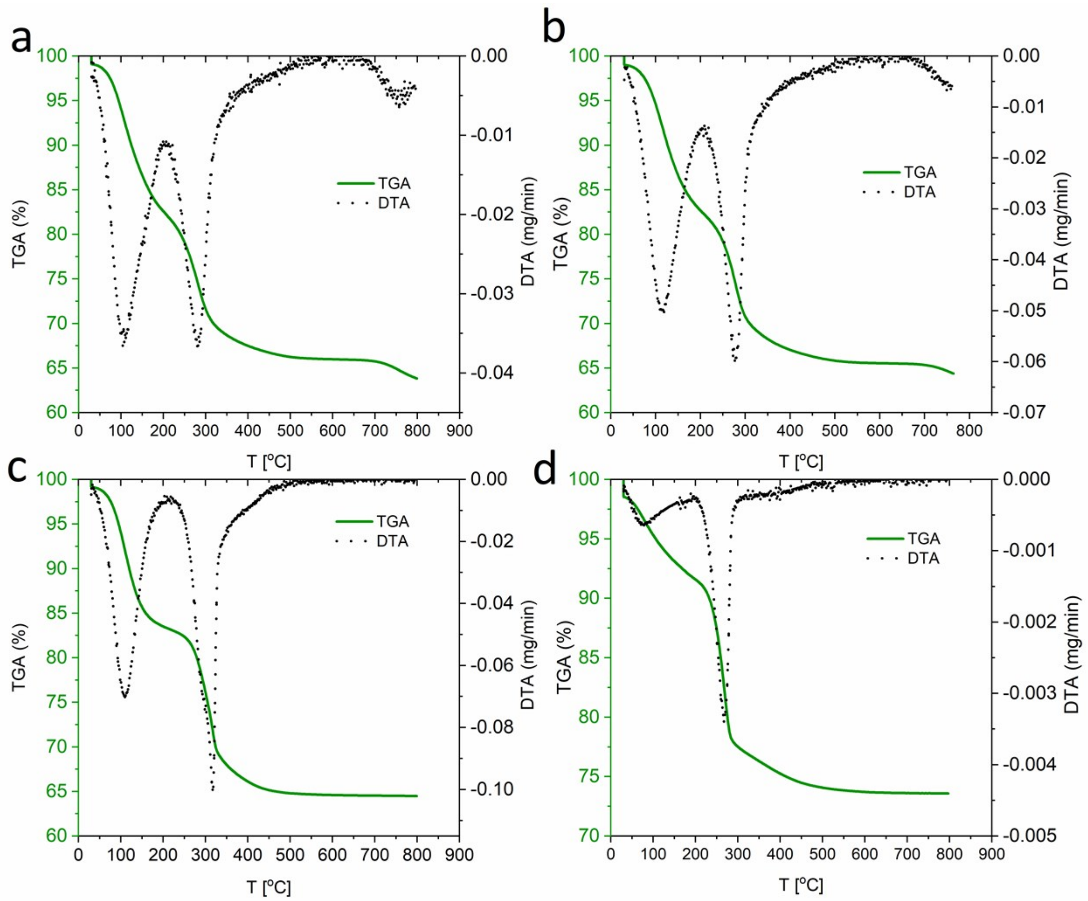

Figure S- 3: TGA and TDA analysis of a) NiFe20, b) NiFe15, c) NiFe7, d) Ni-B materials

The chemical formula of the various doped samples is estimated from TGA (Figure S- 3) and ICP measurements. The TG and DTA curves for the samples are shown in Figure S- 3 and compared to the commercial β-$Ni(OH)_2$. This last sample shows only one weight loss process between 200 °C and 400 °C, characteristic of the nickel hydroxide decomposition into NiO and H_2O. In contrast all three iron doped samples show two weight loss steps between 0 and 600 °C. The first one appearing between 0 and 200 °C corresponds to the loss of adsorbed and intercalated water, the second between 200 °C and 400 °C is due to the dissociation of Fe-$Ni(OH)_2$ into Fe-NiO. The content of adsorbed and intercalated water in the three NiFe-LDH materials corresponds to 18 wt% of the material weight. The beginning of weight loss observed after 700 °C in Figure S- 3 a and b is attributed to sulphate anions present in the sample[2]. This confirms that samples NiFe20 and NiFe15 are alpha phase nickel hydroxides with sulphate anions intercalated. The samples Ni-B but also NiFe7 do not show this decrease because Ni-B is a beta nickel hydroxide with no anions intercalated and NiFe7 is a mixed α/β phase interstratified material with probably not enough sulphate intercalated to be noticed on its TGA graph.

The amount of nickel in the samples, as well as the molar ratio x=Fe/(Ni+Fe) determined by ICP are displayed in Table 2. The molar ratio Fe/(Ni+Fe) is estimated to be 7, 13 and 18 % for NiFe7, NiFe15 and NiFe20 respectively which allows to determine the stoichiometry of Ni and Fe in the material. Sulphate salts being used for the NiFe-LDH synthesis, one can expect SO_4^{2-} to be intercalated in the interlayer space of the crystal of the freshly prepared samples to compensate the excess of positive charges induced by the iron substitution. Thus a proposition of the different materials chemical formula is given in Table S-1.

Several authors suggest that after ageing in KOH, the anions intercalated is being replaced by carbonate anions (in case of use in a battery that is not fully closed to ambient air). Carbonates can indeed also be formed in the KOH solution during its fabrication in ambient air. According to Mendiboure et al.[3] the affinity

of CO_3^{2-} with the nickel oxide layers is stronger than for the sulphate anions, the ion-exchange equilibrium constants following the sequence $CO_3^{2-} > SO_4^{2-} > OH^- > F^- > Cl^- > I^-$. Thus, Hunter et al.[4] show for NiFe-LDH materials prepared with various anions intercalated that, after ageing in KOH in ambient air, the initial intercalated anions were mostly replaced by carbonates. For their NiFe-SO_4^{2-} material, this anion exchange comes with a reduction of the interlayer distance from 8.6-8.2 Å to 7.6 Å. The same observation was made in the present study, with a reduction of the interlayer space from 8.2 Å to 7.7 Å for material NiFe20 after 1 month of ageing in KOH. Once can make the assumption that after the ageing period, carbonates replace sulphates in all the NiFe-LDH samples. Another possibility in the absence of ambient air CO_2 is replacement of sulphates by smaller OH^-. In our experiments we observe the same c-axis reduction for aging in a closed bottle (no CO_2 ingress from air) and aging in a not fully closed battery and electrolysis cell (not fully closed to allow for O_2 and H_2 evolution and occasional water replenishment). Further analysis, such as IR-Spectroscopy, would be necessary to confirm the presence of CO_3^- and OH^-.

**Sample**	**Formula**
NiFe20	$Ni_{0.82}Fe_{0.18}(OH)_2(SO_4^{2-})_{0.09\_}1.2H_2O$
NiFe15	$Ni_{0.87}Fe_{0.13}(OH)_2(SO_4^{2-})_{0.07\_}1.2H_2O$
NiFe7	$Ni_{0.93}Fe_{0.07}(OH)_2(SO_4^{2-})_{0.04\_}1.2H_2O$

Table S-1: Chemical formula of the NiFe-LDH materials directly after synthesis

Electrochemical characterisation

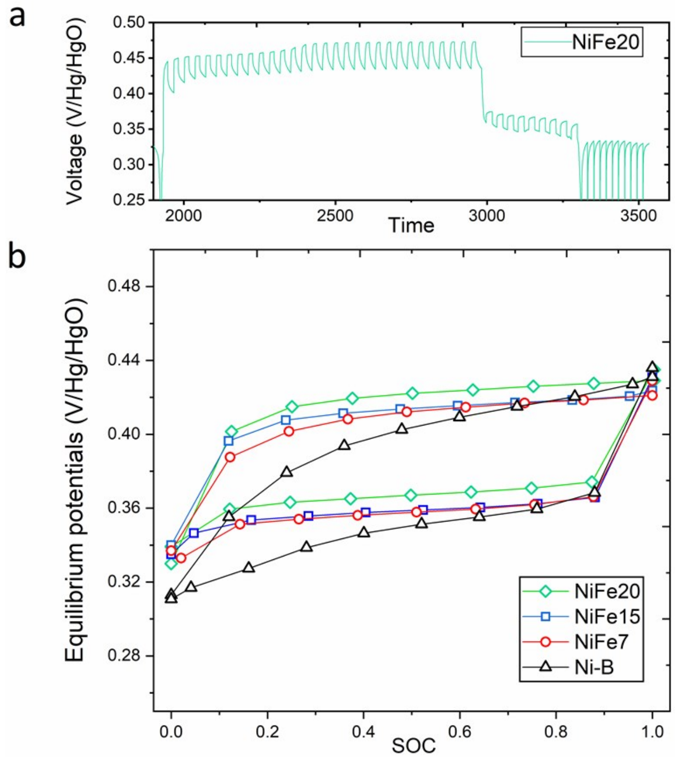

Figure S- 4: GITT measurement of sample NiFe20 a) and evolution of the equilibrium potential with the SOC determined by GITT for sample NiFe20, NiFe15, NiFe7, Ni-B.

The GITT measurements were performed by charging and discharging at 0.2C by steps of 15 minutes, intercalated by a resting period of 20 minutes during which the open circuit potential (O.C.P) was

monitored. For the different state of charge, the equilibrium potential value corresponds to the O.C.P measured at the end of the resting period. Figure S- 4 a shows an example of the GITT measurement performed on NiFe20 and the equilibrium potentials determined by this method in Figure S- 4 b.

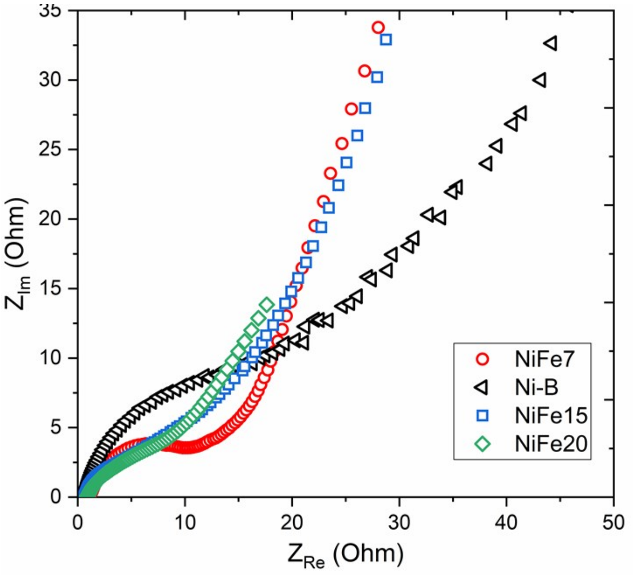

Figure S- 5: Nyquist plots for NiB and the NiFe-LDH electrodes

Energy efficiency calculation

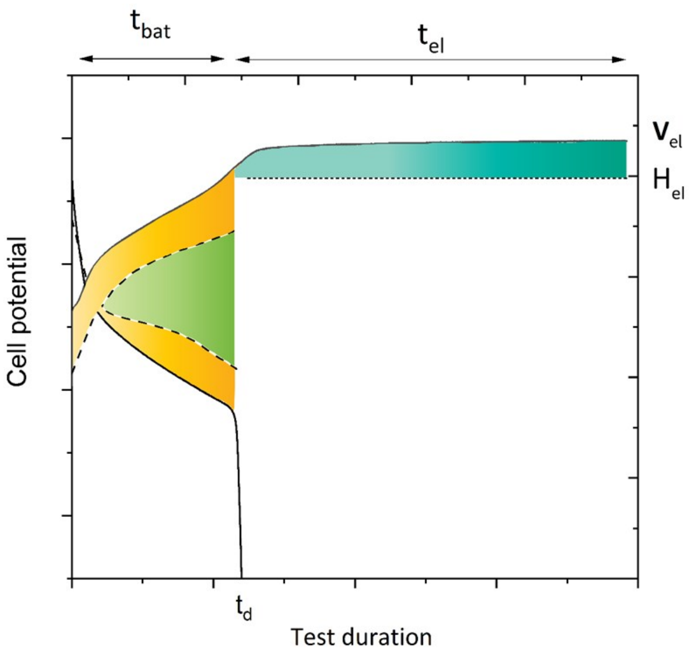

Figure S- 6: Illustration of the various sources of energy loss, orange for the kinetic loss during battery charging, green for the battery hysteresis loss and blue for the electrolysis loss compared to H_{el}, the thermoneutral potential for the OER.

The energy efficiency loss of the hybrid battery electrolyser is represented by the coloured areas in Figure S- 6 and can be expressed as follows:

$$\varepsilon_{total} = \varepsilon_{bat} + \varepsilon_{el} \quad \text{Eq-S 6}$$

$$\varepsilon_{bat} = \frac{\int_{tc}^{tc+td} V_d . I_d . dt}{\int_{0}^{tc} V_c . I_c . dt} \quad \text{Eq-S 7}$$

$$\varepsilon_{el} = \frac{\int_{0}^{tc} H_{el} . I_{el} . dt}{\int_{0}^{tc} V_c . I_C . dt} \quad \text{Eq-S 8}$$

with t_c and t_d the full duration of the charge and discharge respectively, V_c and V_d the charge and discharge potential, I_c, I_d, I_{el} the current inserted for the charge, discharge and electrolysis (here $I_c = I_d = I_{el}$) and H_{el} the thermoneutral potential of water splitting.

The energy efficiency loss *L* ($L = 1 - \varepsilon$) corresponds to the energy loss (in Joule) divided by the total energy inserted and is composed of the battery and electrolysis losses (coloured are in Fig S-3):

$$L_{total} = L_{bat} + L_{el} \quad \text{Eq-S 9}$$

$$L_{bat} = \frac{Loss_{bat}}{Energy_{inserted}} = \frac{(\bar{V}c - \bar{V}d).C_d}{\bar{V}c . C_c} \quad \text{Eq-S 10}$$

$$L_{el} = \frac{Loss_{el}}{Energy_{inserted}} = \frac{(V_{el} - H_{el}).(C_{el})}{\bar{V}c . C_c} \quad \text{Eq-S 11}$$

$\bar{V}c$ is the full cell average voltage for the charge between $t=0$ and $t=t_{bat};$

$$\bar{V}c = \frac{\int_{0}^{tbat} V_c . dt}{\int_{0}^{tbat} t . dt} \quad \text{Eq-S 12}$$

$\bar{V}d$ is the average voltage of the discharge:

$$\bar{V}d = \frac{\int_0^{td} V_d.dt}{\int_0^{td} t.dt}$$ *Eq-S 13*

t_{bat} corresponds to the duration necessary for the charge inserted to equal the discharge capacity, t_{bat}=t_d, t_{el} is the duration of the overcharge, t_{el}=t_c-t_{bat} (Cf Figure S- 6). C_d is the discharged capacity, C_c is the total charge inserted (overcharge included) and C_{el} is capacity inserted during the overcharge (C_{el}=C_c-C_{bat}). V_{el} and H_{el} are the potential of the electrolysis plateau and the thermoneutral potential respectively.

The energy efficiency losses can be disentangled into nickel electrode and iron electrode losses:

$$L_{total} = L_{total}(Ni) + L_{total}(Fe)$$ *Eq-S 14*

$$L_{total}(Ni) = \frac{Loss_{bat}(Ni)}{Energy_{\,inserted}} + \frac{Loss_{el}(OER)}{Energy_{\,inserted}} = L_{el}(Ni) + L_{el}(OER)$$ *Eq-S 15*

$$L_{total}(Ni) = \frac{(\bar{V}c\ (Ni) - \bar{V}d\ (Ni)).C_d}{\bar{V}c\ .C_c} + \frac{(V_{OER} - E_{TN}(OER)).(C_c - C_d)}{\bar{V}c\ .C_c}$$ *Eq-S 16*

E_{TN} (OER) is the thermoneutral potential for the oxygen evolution reaction, V_{OER} the potential of the OER plateau.

$Loss_{bat}(Ni)$ can be also rewritten as a function of its hysteresis and kinetic components (Figure S- 6 green and yellow area respectively):

$$L_{bat}(Ni) = \frac{Loss_{hysteresis}}{Energy_{\,inserted}} + \frac{Loss_{kinetic}}{Energy_{\,inserted}}$$ *Eq-S 17*

$$L_{bat}(Ni) = \frac{(\bar{E}_c(Ni)\ - \bar{E}_d(Ni)) \times C_d}{\bar{V}c\ .C_c} + \frac{(\bar{\eta}_c(Ni) + \bar{\eta}_d(Ni)) \times C_d}{\bar{V}c\ .C_c}$$ *Eq-S 18*

$\bar{E}_c(Ni)$ and $\bar{E}_d(Ni)$ are the averaged equilibrium potentials of the nickel electrode, measured with GITT experiments during the charge and the discharge respectively (Figure S- 4):

$$\bar{E}_c(Ni) = \frac{\int_0^1 OCP_c.dSOC}{\int_0^1 SOC.dSOC}$$ *Eq-S 19*

$$\bar{E}_d(Ni) = \frac{\int_0^1 OCP_d.dSOC}{\int_0^1 SOC.dSOC}$$ *Eq-S 20*

	NiFe20		Ni-B	
	0.1C	4C	0.1C	4C
Loss$_{kinetic}$(Ni) (J)	1.1×10^{-5}	1.6×10^{-5}	2.1×10^{-5}	2.1×10^{-5}
Loss$_{Hysteresis}$(Ni) (J)	1.3×10^{-5}	1.2×10^{-5}	1.8×10^{-5}	1.5×10^{-5}
L_{bat} (%) (Full cell $\bar{V}c$ =1.6 V)	2.3	2.8	2.9	3.3

Table S-2: energy loss, Loss$_{kinetic}$ and Loss$_{Hysteresis}$*, related to kinetic (yellow area in Fig-S-3) and hysteresis (green area in Figure S-6) effects during the nickel electrode charge/discharge process; numeric values for a specific lab test electrode. Estimation of the nickel electrode contribution to the full cell energy efficiency loss, L_{bat}, considering a full cell average charge voltage of 1.6 V.*

	NiFe20	Ni-B
	4C	4C
Loss$_{el}$(OER) (J)	-3.1 $\times 10^{-5}$	-2.6 $\times 10^{-5}$
L_{el}(OER) (%) (Full cell $\bar{V}c$ =1.6 V)	-3.1	-2.4

Table S-3: energy loss, Loss$_{el,}$, related to the OER overpotential (blue area in Figure S- 6) during Ni electrode overcharge. Estimation of the nickel electrode contribution to the full cell energy efficiency loss, L_{el}(OER), considering a full cell average charge voltage of 1.6 V.

Thermoneutral potential of OER:

$$E_{TN} = \frac{-(\Delta G + T\Delta S)}{nF}$$ *Eq-S 21*

$$E_{TN}(OER) = Eeq(OER) - \frac{T\Delta S(OER)}{nF}$$ *Eq-S 22*

$$\Delta S(OER) = 4S(OH^-) - 2S(H_2O) - S(O_2) = -387.9\ J/K.mol$$ *Eq-S 23*

With: $S(O_2) = 205.16 J/K.mol, S(H_2O) = 69.95\ J/K.mol, S(OH^-) = -10.7 J/K.mol$

$$Eeq(OER) = E°(OER) + 0.06 \times \log[OH^-] = 0.353\ V$$ *Eq-S 24*

$$E_{TN}(OER) = 0.653\ V/SHE = 0.601\ V/Hg/HgO$$ *Eq-S 25*

Stability of the NiFe20 material

- **OER stability**

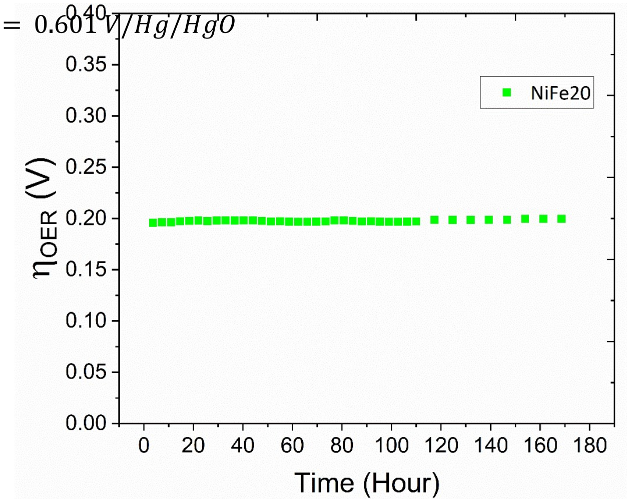

Figure S- 7: OER overpotential stability (iR corrected) over 180 hours of charge at 4C (~4 mA/cm²)

- **SEM analysis**

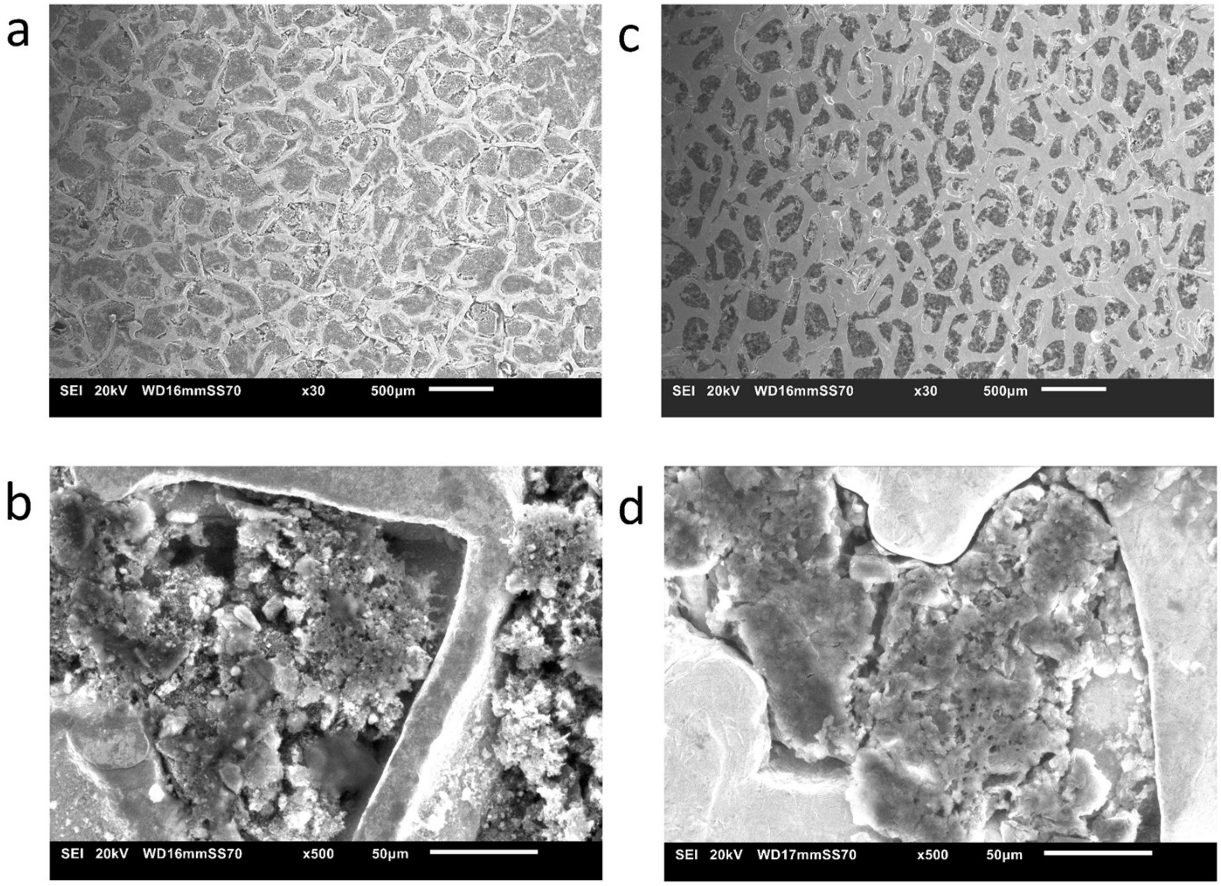

Figure S- 8: SEM picture of a NiFe20 electrode a) and b) fresh electrode at x30 and x500 respectively, c) and d) electrode after 1000 cycles at x30 and x500 respectively.

- **XRD analysis**

XRD analysis is performed on the NiFe20 electrode after 1000 cycles to confirm the stability of the alpha phase. The XRD spectrum of the electrode (Red) in Figure S- 9 shows some high intensity peaks corresponding to different materials such as carbon and nickel used for the electrode making which are not indexed in the figure being not

relevant for the hydroxide. The results show that the material consists mainly of α-$Ni(OH)_2$ with a d003 peak at 13.8°. The presence of γ-$Ni(OH)_2$ is also revealed by a peak at 15.7° overlapping partly with the d003 peak of the alpha phase. This reveals that a part of the active material could not be discharged. The small peak at 22.7° can be assigned to the d001 peak of the β-$Ni(OH)_2$ which suggests that a portion of the nickel hydroxide is converted to β-$Ni(OH)_2$ during the ageing and the cycling of the sample. The area of the peak suggests that the portion of β-$Ni(OH)_2$ is low relatively to the alpha/gamma couple but this could explain the reduction of the capacity from 1.57 e^-/Ni to 1.4 e^-/Ni after the 1000 cycles. A comparison of the NiFe20 electrode with the NiFe20 material aged in KOH highlights a small shift in the d003 and d006 peaks position of the α-$Ni(OH)_2$ reflecting a reduction of the interlayer distance. As mentioned before, this phenomenon could be explained by the pursuit of the intercalated anions replacement with carbonate anions along the electrode cycling in the not hermetically sealed test cell.

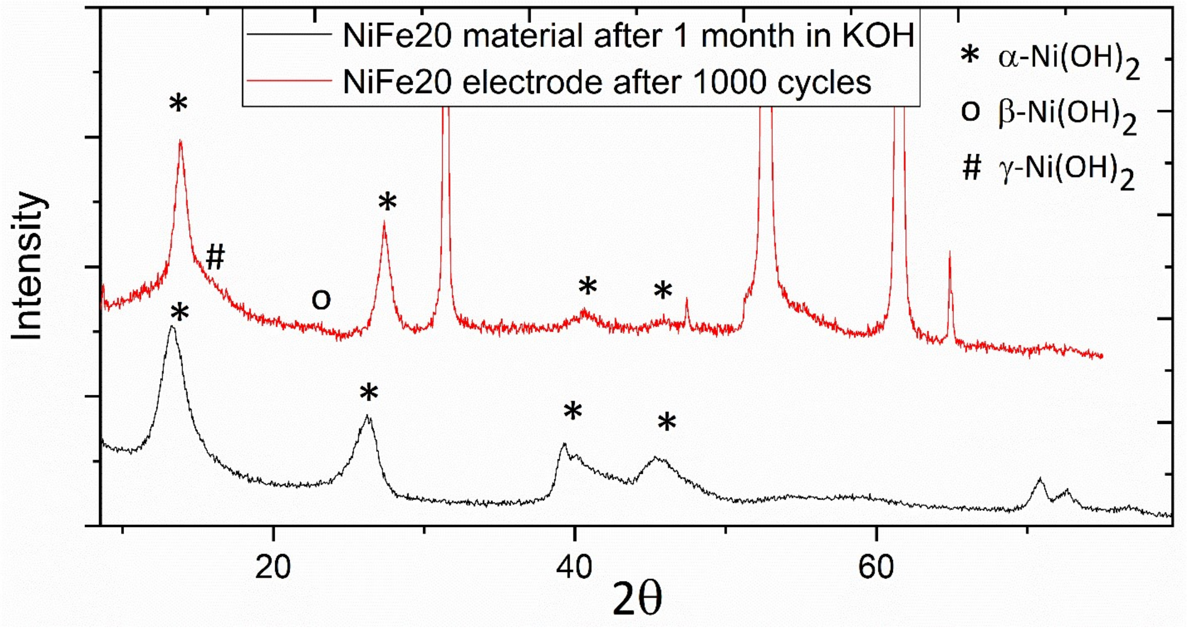

Figure S- 9: XRD spectra of the NiFe20 electrode after 1000 cycles (Red) and of the NiFe20 material aged in KOH for 1 month as comparison (Black). The high narrow peaks in the cycled sample are from the Ni metal current collector.

- **XPS analysis**

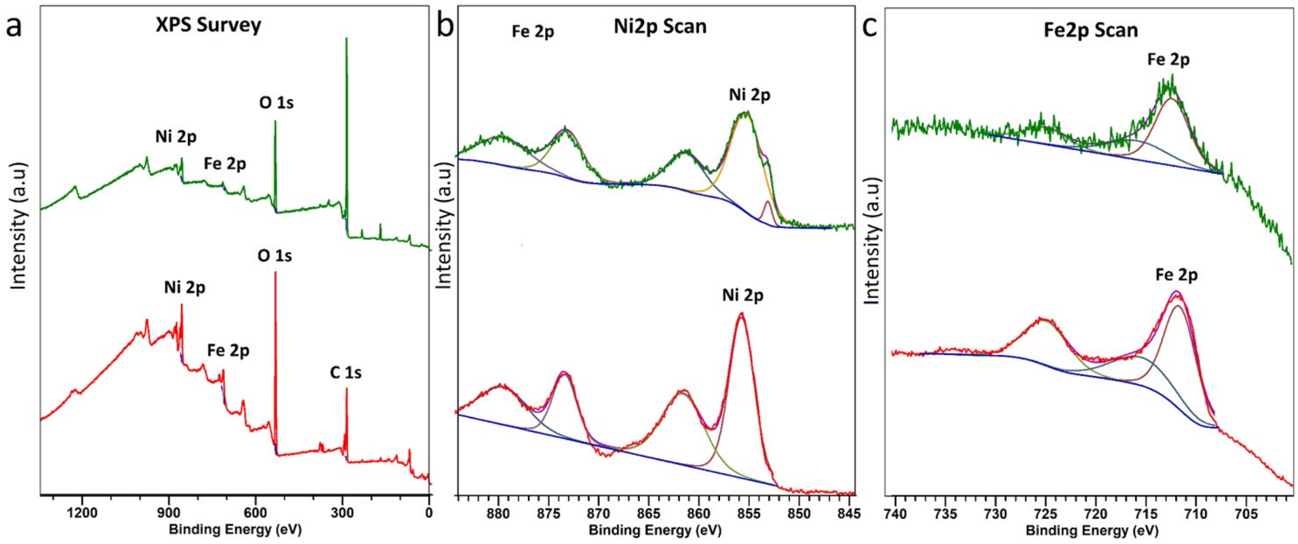

Figure S- 10: XPS spectra of NiFe20 electrodes, in green, for a fresh electrode and in red, for the aged electrode after 1000 cycles. a) Full scan XPS spectra, b) and c) high resolution spectra of the nickel and iron elements respectively.

References

1 F. M. Mulder, B. M. H. Weninger, J. Middelkoop, F. G. B. Ooms and H. Schreuders, *Energy Environ. Sci.*, 2017, **10**, 756–764.

2 A. Raquel, S. Neffer, A. Gomez and G. Fernando, *J. Therm. Anal. Calorim.*, 2019, **140**, 1715–1723.

3 A. Mendiboure and R. Schoellhorn, *Rev. Chim. Min.*, 1986, 819–827.

4 B. M. Hunter, W. Hieringer, J. R. Winkler, H. B. Gray and A. M. Müller, *Energy Environ. Sci.*, 2016, **9**, 1734–1743.
